# Administration of Protein Kinase D1 Induces a Protective Effect on Lipopolysaccharide-Induced Intestinal Inflammation in a Co-Culture Model of Intestinal Epithelial Caco-2 Cells and RAW264.7 Macrophage Cells

**DOI:** 10.1155/2017/9273640

**Published:** 2017-10-26

**Authors:** Ditte Søvsø Gundelund Nielsen, Marlene Fredborg, Vibeke Andersen, Stig Purup

**Affiliations:** ^1^Department of Animal Science, Aarhus University, Blichers Allé 20, 8830 Tjele, Denmark; ^2^Hospital of Southern Jutland, 6200 Aabenraa, Denmark; ^3^Institute of Regional Health Research and Institute of Molecular Medicine, University of Southern Denmark, 5000 Odense, Denmark

## Abstract

Inflammatory bowel diseases (IBD) are chronic inflammatory diseases involving all or part of the gastrointestinal tract. The stress-activated serine-threonine protein kinase D1 (PKD1) protein has previously been implicated in intestinal immune regulation. The objective of this study was to evaluate the effects of human PKD1 in relation to intestinal inflammation, using a co-culture model of intestinal epithelial Caco-2 cells and RAW264.7 macrophages. An inflammatory response was induced in the macrophages by lipopolysaccharide (LPS), upregulating the expression of tumour necrosis factor alpha (TNF-*α*), interleukin- (IL-) 1*β*, and IL-6 besides increasing the secretion of TNF-*α* protein. The effect of administering PKD1 to Caco-2 was evaluated in relation to both amelioration of inflammation and the ability to suppress inflammation initiation. Administration of PKD1 (10–100 ng/ml) following induction of inflammation induced downregulation of TNF-*α* expression in RAW264.7 cells. In addition, PKD1 administered for 3 h prior to LPS stimulation reduced the subsequent inflammatory response through downregulation of TNF-*α*, IL-1*β*, and IL-6 in RAW264.7 cells. These results demonstrate a potential role of PKD1 in the intercellular communication between intestinal epithelial and immune cells, proposing a protective effect of PKD1 on the induction of an inflammatory response in macrophages, an important aspect during the pathogenesis of IBD.

## 1. Introduction

IBD including Crohn's disease (CD) and ulcerative colitis (UC) are characterized by an impaired intestinal barrier function and enhanced intestinal inflammation. IBD has emerged as global diseases with increasing incidence rates specifically in newly industrialized countries [1]. The pathogenesis of IBD is related to a decreased intestinal barrier function, impeding the segregation between the intestinal epithelial cells (IECs) and the intestinal milieu. Specifically, an impaired intestinal barrier function is associated with disease severity in IBD patients [2] as microbes infiltrate the underlying tissue, inducing inappropriate and excessive activation of the mucosal immune system [3]. Indeed, inappropriate responses to these external stimuli by immune cells including dendritic cells and macrophages contribute to the pathogenesis of IBD as immune and tissue homeostasis is disturbed [4]. Reducing inflammation is of utmost importance in IBD patients and specifically proinflammatory mediators such as IL-6 and TNF-*α* are enhanced in both CD and UC [5]. Indeed, the immune-regulatory nuclear transcription factor-kappaB (NF-*κ*B) is strongly activated in IECs and macrophages during the course of IBD, augmenting the secretion of proinflammatory cytokines as TNF-*α*, IL-1, IL-6, IL-12, and IL-23 [6]. However, beneficial effects of the proinflammatory cytokines such as IL-1*β*, IL-6, and TNF-*α* are also observed in relation to both inflammation and reepithelialization of wounded intestinal tissue, emphasizing the importance of temporal control of their expression to balance appropriate responses to intestinal damage and inflammation [7–9].

**Figure 1:**
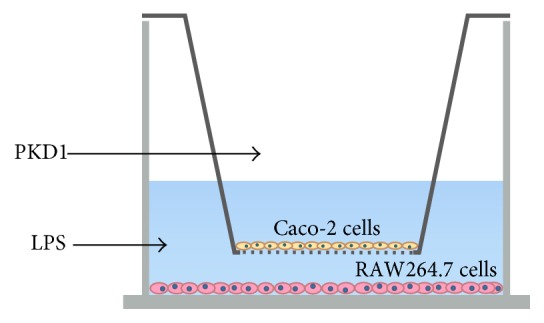
Schematic illustration of the co-culture system. Protein kinase D1 (PKD1) was administered to Caco-2 cells grown for 21 days in transwell inserts, while lipopolysaccharide (LPS) was administered to RAW264.7 macrophage cells to induce an inflammatory response.

Human PKD1 is a stress-activated protein kinase implicated in signal transduction, cell proliferation, differentiation, migration, and immune regulation [10]. PKD1 is expressed in B- and T-cells [11] and activated in RAW264.7 macrophage cells upon recognition of bacterial pathogen DNA, inducing activation of NF-*κ*B and subsequent expression of the proinflammatory cytokines, TNF-*α*, IL-6, IL-8, and IL-12 [12]. Also, PKD1 induces activation of NF-*κ*B in response to oxidative stress [13], protecting IECs against oxidative-stress-induced apoptosis [14]. This suggests an important role for PKD1 in relation to intestinal inflammation. In the present study we investigated two important questions: (1) Can administration of human recombinant PKD1 to IECs ameliorate an already established inflammatory response in macrophages? (2) Can administration of human recombinant PKD1 to IECs suppress initiation of a subsequent LPS-induced inflammatory response in macrophages? To study these effects, we employed an* in vitro* intestinal inflammation model using a co-culture system with intestinal epithelial Caco-2 cells and RAW264.7 macrophage cells (Figure 1) [15].

## 2. Materials and Methods

### 2.1. Cell Lines and Culturing

The human epithelial colorectal adenocarcinoma cell line, Caco-2, was kindly donated by Jan Trige Rasmussen (Aarhus University, Denmark). Cells were cultivated in Dulbecco's Modified Eagle Medium (DMEM, Life Technologies, Denmark) supplemented with 10% fetal calf serum (FCS, Bio-Whittaker, Denmark), HEPES (10 mM, Life Technologies), GlutaMax (2 mM, Life Technologies), and penicillin-streptomycin (1% v/v, Sigma-Aldrich, Denmark). RAW264.7 macrophage cells (ATCC, Germany) were cultivated in DMEM supplemented with 10% FCS, 2 mM GlutaMax, and 1% v/v penicillin-streptomycin. Cells were maintained at 37°C and 5% CO_2_. Caco-2 cells were passaged using 0.05% Trypsin-EDTA (Life Technologies) and RAW264.7 cells were passaged by scraping off cells when reaching 80–90% confluence.

### 2.2. Co-Culture Model

Caco-2 cells (passages 10 to 12) were seeded at a density of 4.76 × 10^5^ cells/cm^2^ in polyethylene terephthalate cell culture inserts with a pore size of 0.4 *μ*m (BD Falcon, VWR, Denmark) and placed in 6-well cell culture plates (BD Falcon, VWR, Denmark). Cells were cultured for 21 days. On day 20, RAW264.7 cells (passages 5 to 8) were seeded in 6-well cell culture plates at a density of 1.04 × 10^5^ cells/cm^2^ in 10% FCS cell culture media. To establish the co-culture model, Caco-2 inserts were transferred to RAW264.7 wells on day 21. RAW264.7 cells were stimulated with 1 *μ*g/ml LPS (*Escherichia coli* O127:B8, Sigma-Aldrich) whereas 0.001, 0.1, 10, and 100 ng/ml PKD1 (129.3 kDa, Thermo Fisher Scientific, Denmark) were added apically to Caco-2 cells (Figure 1). Two studies were performed, either LPS was administered for 3 h followed by PKD1 for 3 h or PKD1 was administered for 3 h followed by LPS for 6 h. Supernatants were collected and stored at −80°C for ELISA analysis, while cells were harvested and stored in RNAlater at −80°C for qPCR analysis. Three biological replicas were performed.

### 2.3. Transepithelial Electrical Resistance

Triplicate measurements were performed using a Millicell-ERS volt-ohm meter combined with a MERSSTX03 electrode (Millipore, Denmark) according to the manufacturer's guidelines. Caco-2 cells were considered fully differentiated once reaching a stable reading of around 700 Ωcm^2^.

### 2.4. Enzyme-Linked Immunosorbent Assay

Quantikine ELISA analysis of murine TNF-*α* was performed on supernatant from RAW264.7 cells. Samples were analysed in duplicate according to the manufacturer's guidelines (R&D systems, AH diagnostics, Denmark). Absorbance was measured at 450 nm and wavelength corrected at 540 nm using an EnVision 2103 Multilabel Reader (PerkinElmer, USA).

### 2.5. Real-Time Reverse-Transcription PCR

Total RNA was isolated and purified using the NucleoSpin RNA II kit according to the manufacturer's guidelines (Macherey-Nagel, Germany). Total RNA concentration and purity was assessed by measuring the absorbance at 260 and 280 nm using a NanoDrop spectrophotometer. Purified RNA was reverse transcribed using oligo(dT) 12–18 primers, random primers, and SuperScript III Reverse Transcriptase (Invitrogen, Denmark). cDNA synthesis was performed at 42°C for 60 minutes followed by reaction inactivation at 70°C for 15 minutes on an Esco Swift MaxPro Thermal Cycler (Holm & Halby, Denmark). cDNA samples were diluted 1 : 10 and standard samples were diluted 1 : 5. For real-time PCR quantitation a standard dilution series of 1 : 4 was prepared and analysed in triplicate, while cDNA samples were analysed in duplicate. The housekeeping gene, hypoxanthine phosphoribosyl transferase (HPRT1), was quantitated by SYBR GREEN PCR master mix (Applied Biosystems, Sweden) using the forward primer: 5′-CAGTCAACGGGCGATATAAAAGTA-3′ and 5′-CCAGTGTCAATTATATCTTCAACAATCAA-3′ as the reverse primer. The other selected genes were analysed by TaqMan™ Gene Expression Assays consisting of predesigned unlabelled PCR primers (Table 1), TaqMan MGB probes labelled with fluorescein amidite (FAM), and TaqMan Universal Master Mix (Applied Biosystems, Sweden). Fluorescence was detected using the Viaa7 Real-Time PCR System (Applied Biosystems, Sweden) with 40 cycles as follows: 1.6°C/s to 95°C held for 15 sec followed by 1.6°C/s to 60°C for 1 min. GAPDH and HPRT1 were chosen as the housekeeping genes for RAW264.7 and Caco-2 cells, respectively.

### 2.6. Statistical Analyses

Data were analysed using the MIXED procedure of Statistical Analysis Software (SAS Institute Inc., USA, version 9.3). Mean Ct values of GAPDH or HPRT1 were used to normalize transcription of the target genes for RAW264.7 and Caco-2 cells, respectively. Real-time RT-PCR data are presented as LSMEANS with 95% confidence intervals, relative to the unstimulated negative control (=1) of replicate experiments. Results from ELISA are presented as LSMEANS ± standard error of the mean (SEM) of replicate experiments. Significant differences were judged at *P* < 0.05.

## 3. Results

### 3.1. PKD1 Stimulates Secretion of TNF-*α* from RAW264.7 Cells

To evaluate the immunological response of PKD1 we employed the already established co-culture model with intestinal epithelial Caco-2 cells and RAW264.7 macrophage cells [15–18]. Caco-2 cells were grown for 21 days until forming a confluent monolayer of enterocyte-like cells [19], at which point a co-culture with RAW264.7 macrophages were established, enabling crosstalk between the two individual cell monolayers (Figure 1). RAW264.7 macrophages were stimulated with LPS at a concentration of 1 *μ*g/ml for 6 h, reaching maximum TNF-*α* secretion within a 24 h period (data not shown). Various concentrations of PKD1 protein were administered apically to Caco-2 cells either before or after stimulation of RAW264.7 cells with LPS. This enabled us to study the ability of PKD1 to ameliorate an already established inflammatory response as well as the ability to suppress initiation of an inflammatory response subsequently induced by LPS.

**Figure 2:**
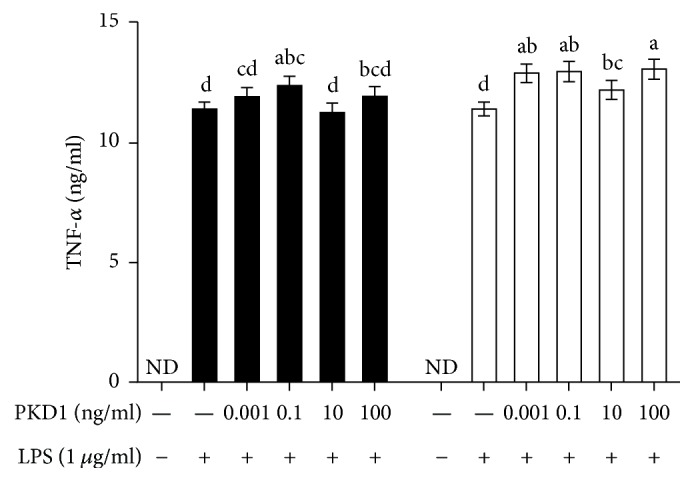
PKD1 stimulate secretion of TNF-*α* from LPS-stimulated RAW264.7 cells. Protein kinase D1 (PKD1; 0.001, 0.1, 10, 100 ng/ml) was administered apically to Caco-2 cells in co-culture with RAW264.7 cells. An inflammatory response was induced in RAW264.7 cells with 1 *μ*g/ml lipopolysaccharide (LPS,* E. coli*: O127). Effects of PKD1 on secretion of TNF-*α* from RAW264.7 cells were evaluated in two different situations: (■) LPS was administered to RAW264.7 cells for 3 h, followed by PKD1 to Caco-2 cells for 3 h to investigate whether PKD1 can ameliorate an already induced inflammation. (□) PKD1 was administered to Caco-2 cells for 3 h at which point RAW264.7 cells were stimulated with LPS for 6 h to evaluate whether PKD1 can suppress inflammation initiation. TNF-*α* level in controls (no LPS stimulation) was undetectable (ND). Data are presented as means ± SEM (*n* = 3). Significant differences (*P* < 0.05) within concentrations of PKD1 are indicated by the lowercase letters.

Stimulation with LPS induced an inflammatory response in RAW264.7 cells within 6 h as observed by secretion of TNF-*α* at a concentration of approximately 11.5 ng/ml into the basolateral compartment (Figure 2). During this 6 h period of LPS stimulation, the integrity of the Caco-2 monolayer remained stable (data not shown). Administration of PKD1 to Caco-2 cells prior to stimulation with LPS induced TNF-*α* secretion at all concentrations (*P* < 0.05). In contrast, PKD1 administered following stimulation with LPS induced TNF-*α* secretion at only 0.1 ng/ml of PKD1.

### 3.2. Effect of PKD1 on the Expression of Inflammatory Markers

**Figure 3:**
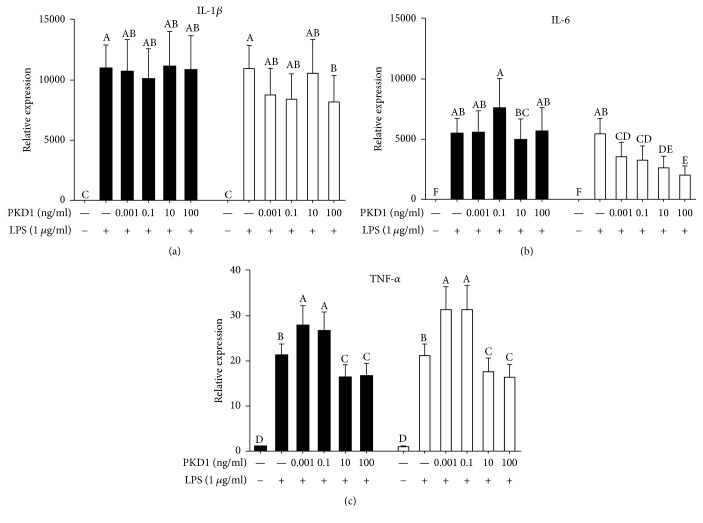
Effect of PKD1 on IL-1*β*, IL-6, and TNF-*α* mRNA expression in RAW264.7 cells. Protein kinase D1 (PKD1; 0.001, 0.1, 10, 100 ng/ml) was administered apically to Caco-2 cells in co-culture with RAW264.7 cells. An inflammatory response was induced in RAW264.7 cells with 1 *μ*g/ml lipopolysaccharide (LPS,* E. coli*: O127). Effects of PKD1 on IL-1*β*, IL-6, and TNF-*α* expression levels were evaluated in two different situations: (■) LPS was administered to RAW264.7 cells for 3 h, followed by PKD1 to Caco-2 cells for 3 h to investigate whether PKD1 can ameliorate an already established inflammation. (□) PKD1 was administered to Caco-2 cells for 3 h at which point RAW264.7 cells were stimulated with LPS for 6 h to evaluate whether PKD1 can suppress inflammation initiation. Data are presented as mean transcriptional levels relative to the negative control (0% FCS, unstimulated) with 95% confidence intervals (*n* = 3). Significant differences (*P* < 0.05) within concentrations of PKD1 and LPS are indicated by the uppercase letters.

To evaluate the effect of the various treatments, mRNA from Caco-2 and RAW264.7 cells were isolated and the expression of selected genes was evaluated by real-time RT-PCR. The inflammatory response induced by LPS in RAW264.7 cells was confirmed by upregulated expression of IL-1*β*, IL-6, and TNF-*α* when compared with the unstimulated control (Figures 3(a)–3(c)). Specifically, a considerable upregulation of both IL-6 and IL-1*β* expression levels was observed (>5,000 times).

Treatment with PKD1 in the LPS-stimulated Caco-2/RAW264.7 co-culture caused alterations in the expression of TNF-*α*, whereas IL-1*β* and IL-6, in RAW264.7 cells, remained unchanged during treatment with PKD1 (Figures 3(a)–3(c)). A concentration-dependent effect on TNF-*α* was observed with low concentrations of PKD1 (0.001–0.1 ng/ml) upregulating TNF-*α* expression and high concentrations of PKD1 (10–100 ng/ml) downregulating the expression of TNF-*α* when compared with the LPS control (*P* < 0.05, Figure 3(c)). Interestingly, the observed effect on TNF-*α* expression levels was independent on the order of PKD1, as a similar concentration-dependent effect was seen, when administering PKD1 prior to LPS stimulation in RAW264.7 cells. Similarly, administration of PKD1 prior to LPS downregulated the expression of IL-1*β* (100 ng/ml) and IL-6 (0.001–100 ng/ml), when compared with the LPS control (*P* < 0.05) in RAW264.7 cells (Figures 3(a) and 3(b)).

**Figure 4:**
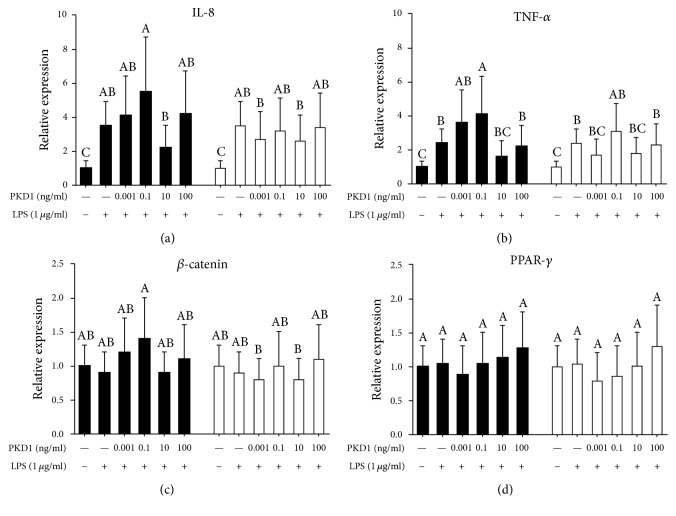
Effect of PKD1 on IL-8, TNF-*α*, *β*-catenin, and PPAR-*γ* mRNA expression in Caco-2 cells. Protein kinase D1 (PKD1; 0.001, 0.1, 10, 100 ng/ml) was administered apically to Caco-2 cells in co-culture with RAW264.7 cells. An inflammatory response was induced in RAW264.7 cells with 1 *μ*g/ml lipopolysaccharide (LPS,* E. coli*: O127). Effects of PKD1 on IL-8, TNF-*α*, *β*-catenin, and PPAR-*γ* expression levels were evaluated in two different situations: (■) LPS was administered to RAW264.7 cells for 3 h, followed by PKD1 to Caco-2 cells for 3 h to investigate whether PKD1 can ameliorate an already established inflammation. (□) PKD1 was administered to Caco-2 cells for 3 h at which point RAW264.7 cells were stimulated with LPS for 6 h to evaluate whether PKD1 can suppress inflammation initiation. Data are presented as mean transcriptional levels relative to the negative control (0% FCS, unstimulated) with 95% confidence intervals (*n* = 3). Significant differences (*P* < 0.05) within concentrations of PKD1 and LPS are indicated by the uppercase letters.

Whereas the inflammatory response initiated in RAW264.7 cells stimulated an immune response in the Caco-2 cells as observed by an upregulated expression of IL-8 and TNF-*α*, only minor differential effects were observed with PKD1 (Figures 4(a)–4(d)). The effect of PKD1 on the expression of IL-8, *β*-catenin, and PPAR-*γ* in Caco-2 cells was nonsignificantly affected, whereas treatment with 0.1 ng/ml of PKD1 after stimulation with LPS upregulated the expression of TNF-*α* when compared with the LPS control in Caco-2 cells (*P* < 0.05).

## 4. Discussion

### 4.1. LPS Stimulated an Inflammatory Response in Caco-2 and RAW264.7 Cells

To evaluate the effect of PKD1 in relation to intestinal inflammation we employed a co-culture model with intestinal epithelial Caco-2 cells and LPS-stimulated RAW264.7 macrophages to resemble gut inflammation, as previously observed [16–18]. Tanoue et al. demonstrated crosstalk between these cells, as LPS-stimulated RAW264.7 cells secrete TNF-*α*, thereby inducing IL-8 mRNA expression in Caco-2 cells [15]. In agreement, we observed LPS-induced secretion of TNF-*α* (11.5 ng/ml) in RAW264.7 cells when RAW264.7 cells were stimulated with 1 *μ*g/ml of LPS (*E. coli* O127, Figure 2). Also, an upregulated expression of TNF-*α*, IL-6, and IL-1*β* was observed in RAW264.7 cells (Figures 3(a)–3(c)). Varying concentrations of LPS have been used for inducing an inflammatory response. Tanoue et al. reported the secretion of 50 ng/ml of TNF-*α* when stimulating with 100 ng/ml of LPS (*E. coli* O127) for 3 h [15], whereas 5 ng/ml of LPS (*E. coli* O127) for 3 h induced secretion of TNF-*α* levels at 350 pg/ml [16]. These differential responses might be explained by varying cell densities as well as different passages of RAW264.7 cells employed in the studies, potentially influencing the responses to LPS stimulation. Concomitantly, LPS stimulation of RAW264.7 cells induced an upregulated expression of IL-8 and TNF-*α* in Caco-2 cells (Figures 4(a)-4(b)). Specifically expression of IL-8 in Caco-2 cells was induced by almost 4-fold when stimulated with 1 *μ*g/ml of LPS, a substantial increase compared to the 2-fold induction observed by Tanoue et al., when stimulating with 100 ng/ml of LPS [15]. Since previous studies have identified Caco-2 cells as unresponsive to LPS [20], these results strongly indicate that RAW264.7 macrophage cells stimulated with LPS induce an inflammatory response in the Caco-2 cells through crosstalk between the two individual cell layers.

### 4.2. PKD1 Displays a Concentration-Dependent Effect in the Amelioration of Intestinal Inflammation

To test the ability of PKD1 protein to ameliorate intestinal inflammation, RAW264.7 cells were stimulated with LPS for 3 h. This was followed by administration of PKD1 to Caco-2 monolayers for 3 h. Whereas no effects were observed on IL-1*β* and IL-6 in RAW264.7 cells, a concentration-dependent effect on TNF-*α* expression levels were observed. Here administration of 10–100 ng/ml of PKD1 downregulated TNF-*α*, whereas lower concentrations (0.001–0.1 ng/ml) caused upregulation of TNF-*α* in RAW264.7 cells. However, a comparable effect was not observed on the secretion of TNF-*α* (Figure 2), indicating that the observed transcriptional effects did not directly translate into corresponding effects at the protein level during this 6 h time period. Since anti-TNF-*α* treatment has emerged as the central target to ameliorate IBD [21], this emphasizes the potential importance of the observed PKD1-induced downregulation of TNF-*α* in RAW264.7 cells. However, PKD1 (0.1 ng/ml) upregulated the expression of TNF-*α* in Caco-2 cells (Figure 4(b)), demonstrating a detrimental effect to the IECs when treated with PKD1. However, due to the limited effects observed in Caco-2 cells, other potential mediators induced upon PKD1 treatment might exert the effects observed in the RAW264.7 cells.

### 4.3. PKD1 Affects the Initiation of Intestinal Inflammation

The ability of PKD1 to hamper inflammation initiation was investigated by administering PKD1 to Caco-2 cells for 3 h prior to a 6 h LPS stimulation in RAW264.7 cells. In RAW264.7 cells, administration of PKD1 to Caco-2 cells suppressed the LPS-induced inflammatory response as IL-1*β* (100 ng/ml PKD1), IL-6 (0.001–100 ng/ml PKD1), and TNF-*α* (10–100 ng/ml PKD1) expression levels were downregulated. Specifically, a concentration-dependent downregulation of IL-6 was observed. The proinflammatory cytokine IL-6 is augmented in acute and chronic wounds [7] and implicated in the course of IBD [22]. This PKD1-dependent downregulation therefore demonstrates a potential important effect of PKD1 in hampering intestinal inflammation. The TNF-*α* response observed in RAW264.7 cells with low PKD1 levels upregulating and high levels downregulating TNF-*α* mRNA levels signifies this concentration-dependent effect of PKD1 observed. Interestingly, secretion of TNF-*α* was induced significantly from the LPS control with no dependency on the concentration of PKD1 (Figure 2). Administration of PKD1 to Caco-2 cells induced TNF-*α* secretion from RAW264.7 cells, while downregulating the mRNA expression at 10–100 ng/ml of PKD1. Hence, the observed effects on the mRNA and protein levels did not directly associate with each other. Since TNF-*α* mRNA and protein levels were evaluated at identical times, these variations might be seen as transcriptional alterations translate into effects at the protein level with a certain delay, limiting the comparability between the two analyses. Interestingly, PKD1 has previously been found to induce the expression of TNF-*α* and IL-6 in RAW264.7 cells upon stimulation with bacterial DNA [12]. The downregulation observed in this study therefore represents a differential effect of PKD1 once administered to the Caco-2 cells prior to initiation of inflammation.

Prostaglandin from LPS-stimulated macrophages induces stimulation of PPAR-*γ* and late activation of IL-10 at 72 h in IECs [23].Here, changes in the TEER (data not shown) and the expression of PPAR-*γ* in Caco-2 cells remained unchanged. Similarly, *β*-catenin, a major component in the formation of adherens junctions (AJs) [24], remained unaffected during the various treatments, despite previous observations of crosstalk between PKD1 and *β*-catenin [25, 26]. Consequently, the studies presented here indicate that a 6 h treatment period with LPS is insufficient for evaluating inflammation-induced effects on *β*-catenin and PPAR-*γ* in Caco-2 cells. Importantly, we did not evaluate the level of PKD1 in the RAW264.7 cell supernatant. The effects observed in the macrophages might therefore be caused by either an indirect or a direct effect, depending on whether PKD1 penetrated the Caco-2 cells and the transwell inserts, entering the lower compartment.

Expression of IL-8 and TNF-*α* in Caco-2 cells was unaffected by the various treatments, implying that either the expression was affected during other time periods or other cellular mediators might be affected by PKD1. Importantly, from these studies it seems that PKD1 has limited effects once an inflammatory response has been initiated. This indicates that the potential anti-inflammatory effect of PKD1 is unable to affect the severe proinflammatory response initiated in RAW264.7 cells upon stimulation with LPS. Oppositely, it seems that a preventive treatment with PKD1 has the ability to hamper a subsequent proinflammatory response induced in the macrophages. However, we have to consider the difference between the periods of PKD1 treatment in the two studies. Whereas the preventive PKD1 treatment was administered for 3 h prior to the 6 h LPS stimulation, giving a total of 9 h treatment with PKD1, Caco-2 cells were only treated with PKD1 for a total of 3 h when studying the ability to ameliorate an already established inflammatory response. Since the inflammation was initiated 3 h prior to the treatment with PKD1, the inflammatory response initiated might be too strong for PKD1 to cause significant effects during a 3 h period.

In conclusion, PKD1 ameliorated an on-going intestinal inflammation in the Caco-2/RAW264.7 co-culture at high concentrations of PKD1, through downregulation of the principal proinflammatory cytokine, TNF-*α*. In contrast, PKD1 administered prior to initiation of an inflammatory response revealed a concentration-dependent anti-inflammatory effect through downregulation of the proinflammatory mediators, IL-6 and TNF-*α*. The studies provide evidence for a potential beneficial and protective effect of PKD1 in relation to intestinal inflammation.

## Figures and Tables

**Table 1:** Primers used during PCR quantitation of selected genes in Caco-2 and RAW264.7 cells.

Analysed in the following	Gene symbol	Gene name	Life-Tech. Id. number	Organism
Caco-2	*TNF-α*	Tumor necrosis factor alpha	Hs01113624_g1	*Homo sapiens*
	*PPARG*	Peroxisome proliferator-activated receptor gamma	Hs01115513_m1	*Homo sapiens*
	*IL-10*	Interleukin 10	Hs00961622_m1	*Homo sapiens*
	*IL-8*	C-X-C motif chemokine ligand 8	Hs00174103_m1	*Homo sapiens*
	*CTNNB1*	Catenin beta 1	Hs00355049_m1	*Homo sapiens*

RAW264.7	*Gapdh*	Glyceraldehyde-3-phosphate dehydrogenase	Mm99999915_g1	*Mus musculus*
	*Tnf-α*	Tumor necrosis factor alpha	Mm00443258_m1	*Mus musculus*
	*Il-6*	Interleukin 6	Mm00446190_m1	*Mus musculus*
	*Il-1β*	Interleukin 1 beta	Mm00434228_m1	*Mus musculus*
